# Synaptic Plasticity Controls Sensory Responses through Frequency-Dependent Gamma Oscillation Resonance

**DOI:** 10.1371/journal.pcbi.1000927

**Published:** 2010-09-09

**Authors:** Se-Bum Paik, Donald A. Glaser

**Affiliations:** 1 Department of Physics, University of California Berkeley, Berkeley, California, United States of America; 2 Department of Molecular and Cell Biology, University of California Berkeley, Berkeley, California, United States of America; University College London, United Kingdom

## Abstract

Synchronized gamma frequency oscillations in neural networks are thought to be important to sensory information processing, and their effects have been intensively studied. Here we describe a mechanism by which the nervous system can readily control gamma oscillation effects, depending selectively on visual stimuli. Using a model neural network simulation, we found that sensory response in the primary visual cortex is significantly modulated by the resonance between “spontaneous” and “stimulus-driven” oscillations. This gamma resonance can be precisely controlled by the synaptic plasticity of thalamocortical connections, and cortical response is regulated differentially according to the resonance condition. The mechanism produces a selective synchronization between the afferent and downstream neural population. Our simulation results explain experimental observations such as stimulus-dependent synchronization between the thalamus and the cortex at different oscillation frequencies. The model generally shows how sensory information can be selectively routed depending on its frequency components.

## Author Summary

In the nervous system, a network of neurons shows interesting population activities. One example is a various frequency of synchronized oscillations which are thought to be important to sensory functions. In particular, it has been reported that gamma frequency rhythms (30∼70Hz) in the cortex can significantly regulate the responses to visual stimuli. In this study, we further investigate the mechanism by which the nervous system can control the effect of gamma oscillation on the modulation of neural responses. We found that the sensory response of the visual cortex strongly depends on the extent of synchronization between external stimulus rhythms and spontaneous gamma oscillations in the cortical network. Furthermore, the simulation results show that the plasticity of the neural circuit can modulate the frequency of spontaneous gamma oscillations, thus readily controlling neural population responsiveness. This finding is related to the question of how the brain efficiently interprets external input signals under various conditions, using its internal neural connectivity. Our study provides insight into this question.

## Introduction

Synchronous oscillations [1]–[3] in neural networks are thought to be important to sensory and cognitive functions [4], [5]. In particular, gamma band oscillations (30∼70Hz) have been observed in various neural circuits [6], [7], and their role has been intensively studied [8]–[11]. Gamma oscillations synchronize the response of neural populations [12], selectively amplify local sensory signals [13], enhance signal transmission by reducing noise [14], and regulate information processing by phase-dependent gating [15]. However, little is known about the mechanism by which the nervous system controls or takes advantage of these gamma oscillation effects. Here we suggest that sensory response can be precisely controlled by the synaptic plasticity of a neural circuit, through the dynamic modulation of spontaneous gamma oscillations. Using a model neural network of the primary visual cortex (V1), we show that (i) the resonance between spontaneous and stimulus-driven oscillations regulates sensory responses and synchrony in a neural population; (ii) the synaptic plasticity of thalamocortical neurons modulates the frequency of spontaneous oscillation in V1; and (iii) this change of spontaneous oscillation regulates gamma resonance, thus controlling the afferent-downstream synchrony. We found that this synaptic modulation can either facilitate or depress the response of the network to stimuli, by changing gamma resonance conditions. Our results suggest that the brain can readily control its synchrony condition for the proper processing of sensory information.

## Results

### Gamma oscillations in model neural network

**Figure 1:**
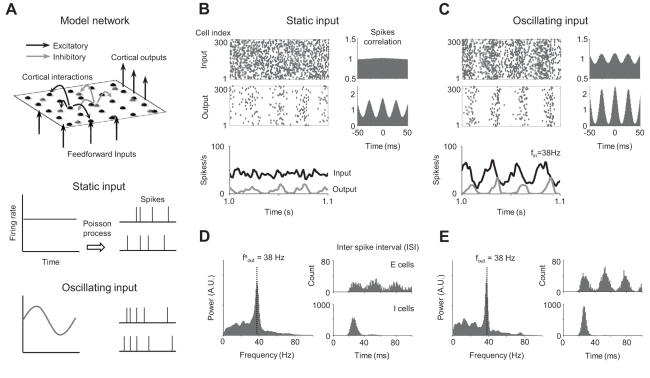
Synchronized population response to static and oscillating inputs. (A) The model visual cortex network. Each excitatory (E) and inhibitory (I) cell receives feedforward inputs from the thalamus, and cortical inputs from the other E and I cells within the range of lateral connections. (B) Population firing rates and spike correlograms for static input and (C) for sinusoidally oscillating input at 38Hz. Correlograms were normalized so that the uncorrelated state is set to unity. (D) Oscillation power spectrum of population firing rate and inter-spike interval (ISI) distribution for static input and (E) for oscillating input. Note that ISI distribution is sharper in (E) than (D), even though gamma oscillation frequencies are the same. For oscillation power spectrum, only the E cells result is displayed, because E and I populations showed identical peak distributions.

We performed our simulations with a model cortical network of excitatory (E) and inhibitory (I) neurons (1mm by 1mm, consisting of 3341 neurons) adapted from our previous study [13] (Fig. 1A, top). When feedforward input spikes (generated by random Poisson process) were injected into the model visual cortex network, neurons generated spontaneous gamma rhythms in their firing pattern (Fig. 1B and C). The spontaneous oscillations were detectable almost whenever the connections between E and I cells were allowed and the input spike rate was above a certain level (∼10spikes/s) that can drive a measurable amount of cortical responses (for detailed parameter tests, see ref. 13). As we reported previously, the frequency of oscillation was modulated by changes in the thalamocortical synaptic strength parameter that controls the excitatory postsynaptic conductance (EPSC) in cortical neurons induced by a feedforward input spike. In the first part of this study, we fixed this thalamocortical synaptic strength and examined the effect of temporal changes in input spike rate only. Subsequently we studied how variations in thalamocorical synaptic strength affect cortical responses.

### Spontaneous and stimulus-driven oscillations

We first controlled input firing rate patterns to examine how gamma oscillation is regulated when feedforward input spike rate varies temporally (Fig. 1A, bottom). For static input, mean input firing rate was set to 40spike/s, and the input spike correlogram indicated no temporal correlation between input spikes (Fig. 1B). Responding to this input, cortical neurons generated an oscillatory output spikes pattern. The oscillation power spectrum showed one strong spontaneous gamma peak at f^s^_out_ = 38Hz; inter-spike interval (ISI) distribution showed that most I cells fired in every gamma cycle, while E cells fired, on average, less than once in a cycle (Fig. 1D). In addition, I cells were synchronized more sharply than E cells in each gamma cycle, indicating that this gamma rhythm was induced by fast-spiking I cells. For oscillating input, in contrast, we drove the network with sinusoidally oscillating input (mean firing rate 40 spikes/s, mean oscillation amplitude ±20 spikes/s), at the same frequency as the spontaneous gamma oscillation frequency for static input (f^s^_out_). In this instance, output oscillation frequency was the same as input frequency (Fig. 1E), but the correlation of output spikes became stronger, and the output rate pattern was phase-locked to input oscillation cycle (Fig. 1C). The ISI distributions of E and I cells were also sharpened, showing that the “gating” or “temporal sharpening” effect [15] of sensory responses is enhanced by coherent input oscillations [16], [17] (Fig. 1E). We refer to this modulation as “resonance” between spontaneous and stimulus-driven oscillations.

### Stimulus-driven oscillations: Input frequency variation

**Figure 2:**
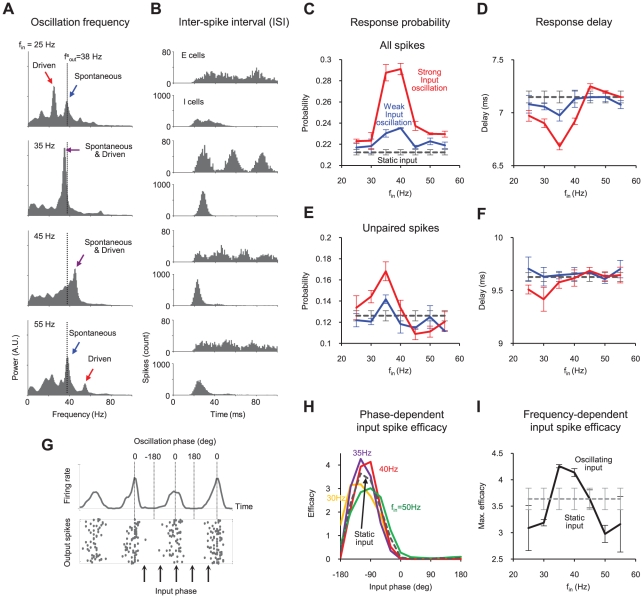
Population response modulation by the resonance between spontaneous and driven gamma oscillations. (A) Cortical output oscillation power spectrum and (B) ISI distributions for sinusoidally oscillating inputs. Note that the resonance between spontaneous and driven oscillations occurs only when input frequency (f_in_) is close to spontaneous gamma frequency (f^s^_out_). (C) Response probability and (D) Response delay to all input spikes of various oscillation frequencies. (E) Response probability and (F) Response delay to temporally “unpaired” input spikes. (G) Relative input timing (phase) in a gamma oscillation cycle. (H) Variation of input spike efficacy by input phase. The efficacy of unpaired input spikes was defined as the relative probability to generate cortical spike, and was measured as a function of input phase. The efficacy was normalized so that the average of each set was set to unity. (I) Maximum input spike efficacy in (H). This shows the network's ability to “gate” or synchronize its output signals.

To further examine the resonance condition between spontaneous and driven oscillations, we injected various frequencies of oscillating inputs to the network. Input frequency was varied within the range f_in_ = 25∼55Hz, similar to the boundary of spontaneous oscillation frequency observed in our previous study [13]. When f_in_ was markedly different from the spontaneous oscillation frequency (f^s^_out_ = 38Hz) for static input, the network displayed two separate peaks in its spectrum (Fig. 2A, red and blue arrows), showing that two different types of oscillations coexist in the cortical response. The spontaneous oscillation peak (blue arrows) remained the same (38Hz) but became weaker than in the previous cases (Fig. 1D, E). The driven oscillation peak (red arrows) appeared at f_in_, confirming that this oscillation was driven by the input spikes pattern. Thus, in this case, spontaneous and driven oscillations existed independently. In contrast, when f_in_ was relatively close but not identical to f^s^_out_, the spontaneous oscillation peak at f^s^_out_ disappeared, and the power spectrum displayed only one peak near f_in_ (Fig. 2A, purple arrows), demonstrating the resonance between spontaneous and driven oscillations. In addition, ISI distributions became sharper than in “irresonant” cases (Fig. 2B). Therefore, when f_in_ is close enough to f^s^_out_, spontaneous oscillation frequency is adjusted close to driven oscillation frequency, and the resonance between two oscillations strengthens cortical gamma rhythm, which enhances the synchronization of cortical spike activities.

### Responsiveness modulation by frequency-dependent gamma resonances

Next, we examined how cortical responsiveness is modulated by gamma oscillation resonance. We varied input frequencies (f_in_ = 25∼55Hz) for different input oscillation strengths. The oscillation amplitude of the input spike rate was set to ±10 spikes/s for weak oscillation and ±20 spikes/s for strong oscillation; mean input rate was 40 spikes/s for all static and oscillating inputs. We measured the output spike probability to a single input spike as the response probability [13] of the cortical neurons. When f_in_ was close to spontaneous oscillation frequency (f^s^_out_ = 38Hz), where the gamma oscillation resonance was strong, response probability was significantly enhanced (Fig. 2C), and response delay was decreased (Fig. 2D). Response modulations were larger for the stronger oscillation. We further investigated whether these modulations might have resulted from temporal correlation changes in the input spike pattern [18], simply due to input frequency variation. In order to remove any influence from input correlation, we sampled temporally “unpaired” input spikes and again measured cortical responses to them. Input spikes were chosen only if there were no other input spikes within 20ms before and after in the same neuron. Even in this case, the response probability was noticeably higher around the gamma resonance area (Fig. 2E), confirming a significant change in cortical responsiveness. However, response delay did not change as much as in the previous result (Fig. 2F), suggesting that this value strongly depends on the temporal correlation of input spikes.

We also examined the dependence of response modulation on the oscillation phase in each cycle. A normalized efficacy of each unpaired input spike was defined as a relative probability to generate a cortical spike, and was measured as a function of input spike phase (Fig. 2G) in each gamma cycle. In Fig. 2H, the input spike efficacy plot shows a “pass” band before 0° phase with a peak value around −90°, and a “block” band after 0° phase, which is known as the mechanism of temporal regulation (or selective gating) of sensory signals in gamma oscillation [15]. In other words, input spikes within the pass band phase have a much higher probability of generating cortical spikes than those within the block band. We found that the pass band was sharpened by gamma resonance. During resonance, the pass band amplitude grew higher and the width narrowed (Fig. 2H, f_in_ = 35Hz and 40Hz). The increase of the maximum in the normalized efficacy means that the pass band is sharpened by gamma resonance (Fig. 2I). As a result, the network's ability to synchronize cortical responses was enhanced by gamma resonance. This result suggests that sensory responses can be manipulated by controlling gamma resonance.

### Spontaneous gamma oscillation frequency modulation by synaptic plasticity

**Figure 3:**
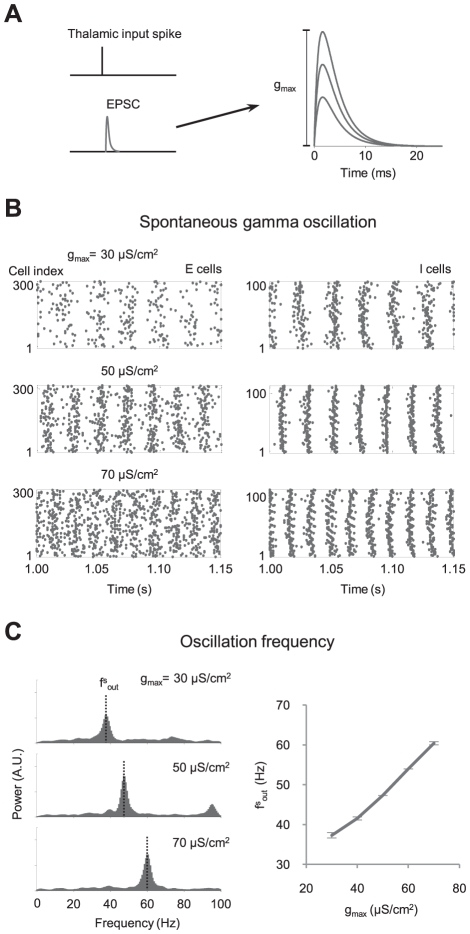
Control of gamma oscillation by synaptic plasticity. (A) The amplitude (g_max_) of excitatory postsynaptic conductance (EPSC) by thalamocortical input spikes is controlled as a simulation of synaptic plasticity. (B) Spontaneous synchrony in the spike firings of E and I cells for various g_max_. (C) Spontaneous gamma oscillation frequency modulation by synaptic plasticity.

It was previously reported that the frequency of gamma oscillation can be rapidly modulated by instantaneous changes in synaptic excitation-inhibition balance [13], [19], [20]. Based on these findings, we hypothesized that the synaptic plasticity of thalamocortical connections can control the frequency of spontaneous cortical gamma oscillation, and therefore regulate sensory responses. To test this idea, we first examined how the frequency of spontaneous cortical oscillation was regulated by the change of thalamocortical synaptic strength. In our simulations, we controlled the amplitude (g_max_) of EPSC driven by each input spike, as a simulation of the synaptic plasticity of LGN-V1 connections (Fig. 3A). We confirmed that the frequency of spontaneous cortical gamma oscillation (f^s^_out_) increased as g_max_ increased (Fig. 3B), a conclusion that was qualitatively observed in our previous simulations [13]. This frequency variation can be explained by the modulation of synaptic excitation-inhibition [19], [20] and response delay [13]. In our simulations, f^s^_out_ varied from 37Hz to 61Hz (Fig. 3C).

### Resonance modulation by synaptic plasticity

**Figure 4:**
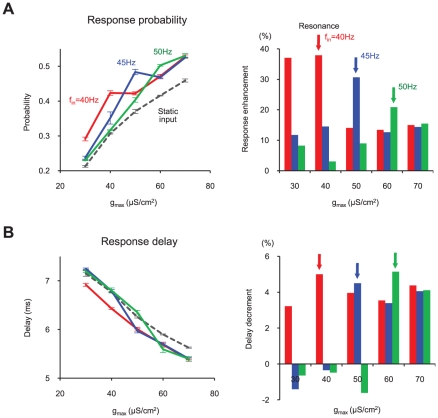
Gamma oscillation resonance tuning by synaptic plasticity. (A) Response probability modulation by frequency-dependent gamma resonance. Response enhancement was defined as the difference between the responses to static input and to other inputs. Note that the peak value of modulation (resonance point) varies by input frequency. (B) Response delay modulation. Negative values in response delay decrement mean increased delay.

Based on these results, we assumed that selective response regulation (depending on input pattern) can be achieved by synaptic plasticity through the control of gamma oscillation resonance, because the resonance frequency of the system is shifted by the changes in g_max_. To further test this assumption, we varied g_max_ under different input frequencies (f_in_ = 40, 45, 50Hz) and measured the response probability of the network (Fig. 4A). As we expected, response probability increased near the gamma resonance region, but the resonance point changed noticeably depending on input frequency. For example, when f_in_ = 40Hz, the response enhancement was largest at g_max_ = 40µS/cm^2^, where g_max_ corresponds to spontaneous oscillation frequency f^s^_out_∼40Hz (Fig. 3C). When f_in_ = 50Hz, the resonance point shifted to g_max_ = 60µS/cm^2^, where f^s^_out_ is close to 50Hz. The response delay of cortical neurons was similarly modulated (Fig. 4B). Gamma resonance thus occurs at different points, depending on input frequency, f_in_, and the amplitude of EPSC, g_max_. In other words, when g_max_ is changed through synaptic plasticity, gamma oscillation regulates the cortical network response selectively, depending on f_in_.

## Discussion

We have shown that sensory responses can be facilitated or depressed, depending on the resonance condition between spontaneous and driven oscillations in the cortex. Here we discuss previous experimental observations that are relevant to our findings. We demonstrate that these experimental results can be explained by our model. We also discuss possible mechanisms by which thalamocortical synaptic plasticity can be controlled by the variation of visual stimuli, which enables the proper modulation of the gamma oscillation resonance and the afferent-downstream synchronization.

### Synchronization between the feedforward and cortical oscillation

Synchronized oscillations of various frequencies are observed in the visual pathway [21]–[23] and are thought to convey information about the visual scene [5], [24]. Previously, Castelo-Branco and colleagues reported a strong correlation of oscillatory responses between the retina, LGN, and the visual cortex in an anesthetized cat [25]. Their observations are in good agreement with our model.

First, cortical oscillation frequencies are clustered as two distinct bands (low-frequency 30–60Hz, high-frequency 60–120Hz). These low- and high- frequency oscillations can coexist in the cortex. High-frequency oscillations in the cortex are shown to be the result of feedforward synchronization with the retina and LGN activity whose oscillation frequencies are in this range. Low-frequency oscillations are shown to be spontaneous gamma oscillations in the cortical circuit. In our model simulation, cortical spikes could be driven by feedforward oscillations of various frequencies, from above spontaneous gamma frequency to over 120Hz (not shown here). Therefore, as shown in our results, cortical responses can be synchronized by two different activities: spontaneous and driven oscillations.

Second, cortical oscillation frequency strongly depends on stimulus condition. For stationary stimuli, cortical neurons are synchronized with high frequency (60–120 Hz) feedforward oscillations. For dynamic stimuli, slow cortical oscillations (30–60Hz) dominate, and subcortical high frequency oscillations become transient. In our model, these two cases are different “resonance” modes; the mode can switch, depending on whether the resonant point is closer to low-frequency cortical gamma oscillation or high-frequency feedforward oscillation. Since the temporal correlation of a feedforward spike train can vary according to stimulus condition, thalmocortical synaptic strength can be modified by activity-dependent short-term plasticity [26]. If this modification arises differently for two stimuli types, the observed variation of cortical oscillation frequency is readily explained. The strengthened synapses increase spontaneous cortical gamma oscillation frequency, resulting in cortical synchronization with the high-frequency feedforward oscillations. On the other hand, if thalamocortical synapses don't change, low-frequency spontaneous gamma oscillations dominate, and the feedforward oscillation becomes transient. As a result of this stimulus-specific synchronization mechanism, cortical activity can be tuned to various oscillation frequencies.

When cortical spontaneous oscillations are strong, LGN activity can be synchronized to cortical oscillations with a retarded phase. Because this situation requires a corticothalamic feedback loop [25], it cannot be fully described by our current model. Generally, this feedback is assumed as a mechanism of population activity normalization or gain control [27]. In our model, the addition of a corticothalamic feedback loop could work as another possible controller of thalamocortical synaptic strength. This mechanism will be further studied in future work.

Another relevant example is that information in the hippocampus is differentially routed, depending on its fast and slow gamma frequency components [28]. This might be a slightly different version of the above mechanism, and we suggest that our gamma resonance model can be a promising candidate for this type of input-specific neuronal synchronization.

### Dynamic resonance tuning by short-term plasticity

Short-term synaptic plasticity [26], [29] has been observed at various places in the nervous system and is thought to be important to the precise tuning of sensory responses depending on stimuli conditions [30], [31]. At the thalamocortical synapses of the somatosensory system, plasticity usually appears as a form of short-term depression [32]–[34]. Similar thalamocortical synaptic depression is also found in the visual system of the cat in vitro [35], [36] and in vivo [37]. However, the effect of thalamocortical synaptic plasticity on visual cortex response is still open to question, because it seems to work both ways: the cortical response can be either depressed or facilitated [18], [38], [39].

From the observations above, it seems possible that stimuli-dependent short-term plasticity at the thalamocortical synapse controls the resonance between feedforward and cortical activities. Rapid changes in synaptic excitation can modulate the frequency and amplitude of gamma oscillations of a neural network [19]. In our simulations, spontaneous gamma frequency varied from 37Hz to 61Hz, which is comparable to measured gamma peak frequency variation in humans [40] caused by excitation-inhibition balance modulation. Since oscillation frequency changes very rapidly in this way, cortical activity can be modulated cycle-by-cycle in gamma rhythms. Thus it can be an effective method of regulating dynamic sensory response to rapidly varying stimuli. In addition, the gamma modulation effect can be spatially localized fairly tightly: a small neural population can be tuned selectively by well-localized feedforward inputs [13]. In this way, a neural network can suitably control its sensory response to complicated (spatially and temporally) visual stimuli patterns.

### Oscillation resonance and long-term plasticity

Although there is no direct experimental evidence yet, it is also possible to relate our model to developing visual systems in young animals, as a tuning mechanism of thalamocortical and corticothalamic synaptic strength. In this case, long-term plasticity [41] becomes important. Long-term potentiation and depression (LTP and LTD) are observed at thalamocortical synapses in the developing somatosensory cortex [42] and visual cortex [43] and are thought to contribute to the stimulus-dependent enhancement of sensory responses. As with the short-term plasticity described above, activity-dependent synaptic plasticity can differentially tune the thalamocortical circuit, depending on the stimulus condition. As a result, the resonance between feedforward and cortical oscillations is modified accordingly, which may contribute to the experience-dependent development of the sensory system. For example, if thalamocortical EPSC varies differentially depending on the visual stimulus pattern by spike timing dependent plasticity (STDP) [44], [45], this will change spontaneous cortical oscillation frequency. LGN-cortex resonance, accordingly, will alternate between two modes: “resonance” and “irresonance.” An experimental observation that cortical plasticity can be driven by different thalamic activity patterns [46] also suggests the possibility of such a resonance control mechanism. Recently it was shown that a single neuron equipped with STDP can robustly detect input spike patterns [47], and that this downstream learning is noticeably facilitated by oscillatory drive with “phase-of-firing coding (PoFC).” Considering that synchronized oscillations are commonly observed in the visual pathway [21]–[23], thalamocortical synapses might be properly learned by oscillations in an earlier pathway, or by activities in the corticothalamic feedback loop.

In conclusion, we demonstrate that the resonance between spontaneous and driven gamma oscillations can significantly regulate sensory responses in a neural population. The synaptic plasticity of thalamocortical neurons can readily control gamma resonance by varying the frequency of spontaneous oscillation, and therefore can selectively enhance or degrade the network's processing of information. Our results suggest a general model of how the nervous system can make use of its internal plasticity for the effective control of sensory responses under various conditions. The simplicity and the wide applicability of our model make it a serious candidate for further experimental tests.

## Methods

### Network model

A two-dimensional layer model of the cortex neural network was used in our simulations, slightly adapted from our previous work [13]. The network size is 1mm by 1mm, including 3341 neurons. The network consists of simplified E (75%) and I (25%) model neurons with Hodgkin-Huxley type Na^+^ and K^+^ ion channels and synaptic conductance channels.

The membrane potential of an individual neuron, 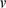, is determined by 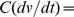 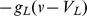 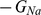 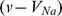 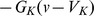 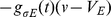 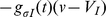 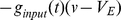, where *σ* is the type of neuron (E or I), *C* is the membrane capacitance, and *g_L_* is the leakage conductance. *g_σE_* and *g_σI_* are the synaptic conductances, providing the cortical E and I inputs. We used the commonly accepted values for physiological parameters (*C* = 10^−6^ Fcm^−2^, *V_L_* = −70mV, *V_Na_* = 55mV, *V_K_* = −80mV, *V_E_* = 0mV, *V_I_* = −80mV and *g_L_* = 50*10^−6^ Scm^−2^). The Hodgkin-Huxley ion channel conductance *G_Na_* and *G_K_* takes the generally known form [48], [49] as in our previous work [13].

We have assumed spatially isotropic local cortico-cortical connections. A neuron's synaptic conductance is given by 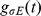 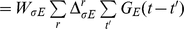, 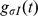 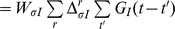, where 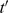
s are input spike timings. The spatial connection factor takes the form 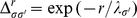, where *r* is the cortical distance, and *σ* and *σ*′ are the type of connected neurons (E or I). The spatial connection decay constants were set as 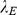 = 200µm, 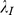 = 100µm. The excitatory and inhibitory postsynaptic conductance fluctuations were set as 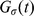 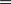 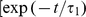 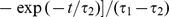. The time constants 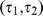 in milliseconds were set as (3, 1) for σ = E and (7, 1) for σ = I. The contribution of each cortical interaction was controlled by weighting factor W*_σσ′_* for the type of neuron pair (*σ*, *σ′*).

The EPSC driven by thalamocortical feedforward input spikes was given by 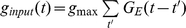. 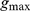 sets the maximum fluctuation amplitude and was varied within 30∼70 µS/cm^2^ as a simulation of synaptic plasticity.

### Simulation and data analysis

Our simulations were performed using the GENESIS 2.3 environment (Text S1) [49]. Simulation outputs were analyzed using Matlab scripts.

## Supporting Information

Text S1 GENESIS simulator configuration: A two-dimensional model neural network.
(0.11 MB PDF)
