# Supplementary material for: Synaptic Plasticity Controls Sensory Responses through Frequency-Dependent Gamma Oscillation Resonance
Source: PLoS Comput Biol. 2010 Sep 9;6(9):e1000927. doi: 10.1371/journal.pcbi.1000927 (PMC2936516; doi:10.1371/journal.pcbi.1000927)
Supplement: Text S1 — GENESIS simulator configuration: A two-dimensional model neural network. (0.11 MB PDF) [file pcbi.1000927.s001.pdf]

Text S1.

GENESIS simulator configuration: A two-dimensional model neural network.

```
// genesis

setglobal TMAX 2000              // simulation time (msec)
setglobal TMID 200               // input stimuli injection time (msec)


//===========================================================================
//                    MAIN SCRIPT - RFS
//===========================================================================


makenetwork      // make a single cell & a cell network
makeinputs       // make input stimuli
makesyncon       // connect cells


//===========================================================================
// variables

// cortical connections
float syn_value = 3      // cortical synaptic connection strength
float EE_w = 10          // WEE relative synaptic connection strength
float EI_w = 40          // WEI
float IE_w = 80          // WIE
float II_w = 10          // WII

float decay              // decay constant for cell communication
int exc_c = 10           // Exc layer connection depth
int inh_c = 5            // Inh layer connection depth

// thalamocortical connections
float sE_w = 30          // thalamocortical synaptic connection strength for E cells
float sI_w = sE_w        // for I cells

// Input stimuli parameters
Float bg_rate = 1                // mean background firing
float s_rate = 40                // mean input spike rate
float s_frequency = 30           // input spike frequency for sinusiodally varing rate (Hz)
float s_rate_amp_e = 20          // input spike rate amplitude for E cells
float s_rate_amp_i = s_rate_amp_e// for I cells



// include in the loops for parameter variation

setglobal WEE {{syn_value} *0.01*EE_w *0.1}
setglobal WEI {{{syn_value} *0.01}*EI_w *0.1}
setglobal WIE {{{syn_value} *0.01}*IE_w *0.1}
setglobal WII {{{syn_value} *0.01}*II_w *0.1}

setglobal WSE {{sE_w}*0.0001}
setglobal WSI {{sI_w}*0.0001}
setglobal WSE_AMP {{sE_w_amp}*0.0001}

setglobal CONN_DECAY {decay*0.01}
setglobal EXC_CONN_DEPTH {exc_c}
setglobal INH_CONN_DEPTH {inh_c}

setglobal D_SIZE 10
setglobal D_SIZE_INH {round {dot_r/{sqrt {RATIO_EI}}}}


setglobal V_RATE_MEAN {s_rate}
setglobal V_RATE_AMP_E {s_rate_amp_e}
setglobal V_RATE_AMP_I {s_rate_amp_i}
setglobal V_RATE_FREQUENCY {s_frequency}
```

```
// firing rate varing (sinusoidally) dot
sine_wd_input

reset
step {TMID} -time
step {TMAX-TMID} -time



//===========================================================================
//                     makenetwork & makesyncon
//===========================================================================

function makenetwork

makeneuron /unitcell {SOMA_L} {SOMA_D}            // in cell.g

create neutral /V1

createmap  /unitcell/soma /V1/exc_layer \
   {MY_NX} {MY_NY}    \
   -delta {V1_SEPX} {V1_SEPY}    \
   -origin {-MY_NX * V1_SEPX / 2} {-MY_NY * V1_SEPY / 2}
setfield /V1/exc_layer z {-V1_SEPZ * 0.5}

createmap  /unitcell/soma /V1/inh_layer \
   {round {MY_NX/{sqrt {RATIO_EI}}}} {round {MY_NY/{sqrt {RATIO_EI}}}}    \
   -delta {V1_SEPX*{sqrt {RATIO_EI}}} {V1_SEPY*{sqrt {RATIO_EI}}}    \
   -origin {-MY_INH * {V1_SEPX*{sqrt {RATIO_EI}}} / 2 } {-MY_INH * {V1_SEPY*{sqrt
{RATIO_EI}}} / 2 }
setfield /V1/inh_layer z {V1_SEPZ * 0.5}

delete /unitcell

end


function makesyncon

   planarconnect /V1/exc_layer/soma[]/spike /V1/exc_layer/soma[]/Ex_channel \
   -relative \      // Destination coordinates are measured relative to source
   -sourcemask box -100 -100  100  100 \   // Larger than source area ==> all cells
   -destmask ellipse 0 0 {V1_SEPX*(EXC_CONN_DEPTH+0.1)}  {V1_SEPY*(EXC_CONN_DEPTH+0.1)} \
   -desthole box {-V1_SEPX*0.5} {-V1_SEPY*0.5} {V1_SEPX*0.5} {V1_SEPY*0.5} \ //exclude self-
connections
   -probability {CXN_PROB}

   planarconnect /V1/exc_layer/soma[]/spike /V1/inh_layer/soma[]/Ex_channel \
   -relative \      // Destination coordinates are measured relative to source
   -sourcemask box -100 -100  100  100 \   // Larger than source area ==> all cells
   -destmask ellipse 0 0 {V1_SEPX*(EXC_CONN_DEPTH+0.1)}  {V1_SEPY*(EXC_CONN_DEPTH+0.1)} \
   -probability {CXN_PROB}

   planarconnect /V1/inh_layer/soma[]/spike /V1/exc_layer/soma[]/Inh_channel \
   -relative \      // Destination coordinates are measured relative to source
   -sourcemask box -100 -100  100  100 \   // Larger than source area ==> all cells
   -destmask ellipse 0 0 {V1_SEPX*(INH_CONN_DEPTH+0.1)}  {V1_SEPY*(INH_CONN_DEPTH+0.1)} \
   -probability {CXN_PROB}

   planarconnect /V1/inh_layer/soma[]/spike /V1/inh_layer/soma[]/Inh_channel \
   -relative \      // Destination coordinates are measured relative to source
   -sourcemask box -100 -100  100  100 \   // Larger than source area ==> all cells
   -destmask ellipse 0 0 {V1_SEPX*(INH_CONN_DEPTH+0.1)}  {V1_SEPY*(INH_CONN_DEPTH+0.1)} \
   -desthole box {-V1_SEPX*0.5} {-V1_SEPY*0.5} {V1_SEPX*0.5} {V1_SEPY*0.5} \ //exclude self-
connections
   -probability {CXN_PROB}


planardelay /V1/exc_layer/soma[]/spike -radial {PROP_SPEED}
planardelay /V1/inh_layer/soma[]/spike -radial {PROP_SPEED_INH}

syndelay /V1/exc_layer/soma[]/Ex_channel {SYN_DELAY}
syndelay /V1/exc_layer/soma[]/Inh_channel {SYN_DELAY_INH}
syndelay /V1/inh_layer/soma[]/Ex_channel {SYN_DELAY}
```

```
syndelay /V1/inh_layer/soma[]/Inh_channel {SYN_DELAY_INH}

end


// cell.g

function makeneuron(path, soma_l, soma_d)
   str path
   float soma_l, soma_d

   float active_area = soma_l*PI*soma_d*1.0

   create neutral {path}
   pushe {path}

   makecompartment soma {soma_l} {soma_d} {Eleak}
   position soma I I R{-soma_l/2.0}
   setfield soma initVm {EREST_ACT}  // properly initialize Vm on reset

   make_hhNa soma hhNa_channel {active_area} {ENa} {EREST_ACT}
   make_hhK soma hhK_channel {active_area} {EK} {EREST_ACT}

   makechannel soma Ex_channel {EEx} {active_area} 3.0 1.0 {GEx}
   makechannel soma Ex_channel2 {EEx} {active_area} 3.0 1.0 {GEx}
   makechannel soma Inh_channel {EInh} {active_area} 7.0 1.0 {GInh}

   create spikegen {path}/soma/spike
   setfield {path}/soma/spike \
        thresh          -20 \           // mV
        abs_refract     5 \      // msec
        output_amp      1
   addmsg {path}/soma {path}/soma/spike INPUT Vm

   pope
end



// channel.g

function makechannel(compartment, channel, Ek, activearea, tau1, tau2, gmax)
   str compartment
   str channel
   float activearea    // cm^2
   // mV
   float Ek
   // msec
   float tau1, tau2
   // mS
   float gmax

   create synchan {compartment}/{channel}
   setfield ^ Ek {Ek} tau1 {tau1} tau2 {tau2} gmax {gmax*activearea}
   link_channel2 {compartment}/{channel} {compartment}
end

function make_hhNa(compartment, channel, activearea, ENa, EREST_ACT)
   str compartment
   str channel
   float activearea    // cm^2
   float ENa, EREST_ACT   // mV

   create tabchannel {compartment}/{channel}
   setfield ^ Ek {ENa} Gbar {120.0*activearea} Xpower 3 Ypower 1 Zpower 0

   setupalpha {compartment}/{channel} X {0.1*(25.0 + EREST_ACT)} -0.1  \
         -1.0 {-1.0*(25.0 + EREST_ACT)} -10.0  \
         4.0 0.0 0.0 {-1.0*EREST_ACT} 18.0 -range -100.0 50.0

   setupalpha {compartment}/{channel} Y 0.07 0.0 0.0  \
         {-1.0*EREST_ACT} 20.0 1.0 0.0 1.0  \
         {-1.0*(30.0 + EREST_ACT)} -10.0  -range -100.0 50.0

   addmsg {compartment} {compartment}/{channel} VOLTAGE Vm
```

```
   addmsg {compartment}/{channel} {compartment} CHANNEL Gk Ek
end

function make_hhK(compartment, channel, activearea, EK, EREST_ACT)
   str compartment
   str channel
   float activearea
   float EK, EREST_ACT   // mV

   create tabchannel {compartment}/{channel}
   setfield ^ Ek {EK} Gbar {36.0*activearea} Xpower 4 Ypower 0 Zpower 0

   setupalpha {compartment}/{channel} X {0.01*(10.0 + EREST_ACT)} -0.01  \
          -1.0 {-1.0*(10.0 + EREST_ACT)} -10.0 0.125 0.0 0.0  \
          {-1.0*EREST_ACT} 80.0 -range -100.0 50.0

   addmsg {compartment} {compartment}/{channel} VOLTAGE Vm
   addmsg {compartment}/{channel} {compartment} CHANNEL Gk Ek
end



// compartment.g

function makecompartment(path, l, d, Erest)
   str path
   float l, d
   float Erest
   float area = l*PI*d
   float xarea = PI*d*d/4
   /* global variables defined in constants.g
   float rm                              // Kohm-cm^2
   float cm                              // uF/cm^2
   float ra                              // Kohm-cm
*/
   create compartment {path}
   // mV
   // Kohm
   // uF
   // Kohm
   setfield {path} Em {Erest} Rm {rm/area} Cm {cm*area} Ra {ra*l/xarea}
end


//===========================================================================
//                     makeinputs
//===========================================================================

create neutral /input

create randomspike /input/randomspike
setfield /input/randomspike min_amp 1 max_amp 1 rate 0 \
        reset 1 reset_value 0 abs_refract 0.1

// creating a plane (map) of input units
// based on the /input

create neutral /LGN

// Independent inputs
// input for exc layer cells
createmap /input /LGN/exc \
               {MY_NX}  {MY_NY} \
                -delta  {V1_SEPX} {V1_SEPY}   \
                -origin {-MY_NX * V1_SEPX / 2} {-MY_NY * V1_SEPY / 2}
setfield /LGN z {-V1_SEPZ * 1}

// input for inh layer cells
createmap /input /LGN/inh \
   {round {MY_NX/{sqrt {RATIO_EI}}}} {round {MY_NY/{sqrt {RATIO_EI}}}}    \
   -delta {V1_SEPX*{sqrt {RATIO_EI}}} {V1_SEPY*{sqrt {RATIO_EI}}}    \
   -origin {-MY_INH * {V1_SEPX*{sqrt {RATIO_EI}}} / 2 } {-MY_INH * {V1_SEPY*{sqrt
{RATIO_EI}}} / 2 }
setfield /LGN z {V1_SEPZ * 1}
```

```
delete /input

// synaptic connection from LGN to V1

// connect independent inputs
   planarconnect /LGN/exc/input[]/randomspike /V1/exc_layer/soma[]/Ex_channel2 \
   -relative \      // Destination coordinates are measured relative to source
   -sourcemask box -100 -100  100  100 \
   -destmask box {-V1_SEPX*0.5}  {-V1_SEPY*0.5} {V1_SEPX*0.5}  {V1_SEPY*0.5} \
   -probability {L_CXN_PROB} // set probability > 1 to connect to all in destmask

   planarconnect /LGN/inh/input[]/randomspike /V1/inh_layer/soma[]/Ex_channel2 \
   -relative \      // Destination coordinates are measured relative to source
   -sourcemask box -100 -100  100  100 \
   -destmask box {-V1_SEPX*{sqrt {RATIO_EI}}*0.5}  {-V1_SEPY*{sqrt {RATIO_EI}}*0.5}
{V1_SEPX*{sqrt {RATIO_EI}}*0.5}  {V1_SEPY*{sqrt {RATIO_EI}}*0.5} \
   -probability {L_CXN_PROB} // set probability > 1 to connect to all in destmask

// synaptic channel weighting
// independent inputs
   planarweight /LGN/exc/input[]/randomspike /V1/exc_layer/soma[]/Ex_channel2 \
      -fixed {WSE}
   planarweight /LGN/inh/input[]/randomspike /V1/inh_layer/soma[]/Ex_channel2 \
      -fixed {WSI}


useclock /LGN/exc/input[]/randomspike 0
useclock /LGN/inh/input[]/randomspike 0


// sine_wd_input.g
// Input spikes (oscillating rate)

function set_in_rate
  if ({getstat -time}>{TMID})

   int i,j
   float sine_input_rate_e
   float sine_input_rate_i
   sine_input_rate_e = { max { { {V_RATE_AMP_E} * { sin { {{getstat -time} - {TMID}} * 2 *
{PI} * {V_RATE_FREQUENCY} / 1000 -{{PI}/2} } } } + {V_RATE_MEAN} } 0 }
   sine_input_rate_i = { max { { {V_RATE_AMP_I} * { sin { {{getstat -time} - {TMID}} * 2 *
{PI} * {V_RATE_FREQUENCY} / 1000 -{{PI}/2} } } } + {V_RATE_MEAN} } 0 }

   for (i= -D_SIZE; i<= D_SIZE; i=i+1)
      for (j= -D_SIZE; j<= D_SIZE; j=j+1)
         if (i*i + j*j <= D_SIZE*D_SIZE)
            setfield /LGN/exc/input[{{MID_CELL}+{MY_NX}*j+i}]/randomspike \
                  rate {sine_input_rate_e/1000} abs_refract 0.1
         end
      end
   end

   for (i= -D_SIZE_INH; i<= D_SIZE_INH; i=i+1)
      for (j= -D_SIZE_INH; j<= D_SIZE_INH; j=j+1)
         if (i*i + j*j <= D_SIZE_INH*D_SIZE_INH)
            setfield /LGN/inh/input[{{MID_CELL_INH}+{MY_INH}*j+i}]/randomspike \
                  rate {sine_input_rate_i/1000} abs_refract 0.1
         end
      end
   end

  end
end


create script_out /LGN/set_in_rate_script
setfield /LGN/set_in_rate_script command set_in_rate
useclock /LGN/set_in_rate_script 2



//==========================================================================
//                      constants
//==========================================================================
```

```
// constants.g

// simulation time step in msec
setclock 0 0.05          // main simulation clock (ms)
setclock 1 1.0           // output interval
setclock 2 1.0           // output interval 2 - for external input update
setclock 3 5.0           // output interval 3 - for random input rate update
floatformat %.7g // reasonable rounding for display


// simulation time and simulation name
addglobal int TMAX                      // simulation time (msec)
addglobal int TMID                      // input stimuli injection time (msec)
addglobal str FILENAME Default          //
addglobal int FIX_RNDSD                 // random number seed

//network size and spacing variables

addglobal float V1_SEPX 0.002           //
addglobal float  V1_SEPY 0.002          //
addglobal float  V1_SEPZ 0.002          //

addglobal int MY_NX  50          // 50-by-50 array
addglobal int MY_NY  50
addglobal float  RATIO_EI 3             //ratio of number of excitatory to inhibitory cells

addglobal int MID_CELL {trunc {MY_NX*{trunc {MY_NY/2}}+MY_NX/2}}   // cell in middle of array
addglobal int MY_INH {round {MY_NX/{sqrt {RATIO_EI}}}}             // size of inhibitory array
        // default setup = 29
addglobal int MID_CELL_INH  {trunc {MY_INH*{trunc {MY_INH/2}}+MY_INH/2}}   // middle cell


//network connectivity variables
//connection depths defined in terms of spacing between excitatory cells

addglobal float  EXC_CONN_DEPTH  10      // depth of connection
addglobal float  INH_CONN_DEPTH  5       // depth of connection
addglobal float  PROP_SPEED  0.01        //speed of axonal propagation (cm/ms) : 0.1 m/s
addglobal float  PROP_SPEED_INH  0.1     //speed of axonal propagation (cm/ms) : 1 m/s

addglobal float  CXN_PROB 1              //probability of connection of neighboring cells
addglobal float  CONN_DECAY 1            //rate of exponential falloff with distance

/* connection strength between cells of various types. WXY denotes the strength
of a synapse from cell type X to cell type Y. */
addglobal float  WEE  10
addglobal float  WEI  40
addglobal float  WIE  80
addglobal float  WII  10

addglobal float  SYN_DELAY  0            // exc synaptic connection delay
addglobal float  SYN_DELAY_INH  0        // exc synaptic connection delay

/* connection strength between LGN and exc, inh layer cells */
addglobal float WSE
addglobal float WSE_AMP
addglobal float WSI
addglobal float L_CXN_PROB 1     //probability of connection from LGN to V1

addglobal float  NOISE_CURRENT_MEAN 0.            // noise current mean
addglobal float  NOISE_CURRENT_RANGE 0.           // noise current standard deviation
addglobal float  NOISE_CURRENT_MEAN_INH 0.        // noise current mean inh
addglobal float  NOISE_CURRENT_RANGE_INH 0.       // noise current standard deviation inh

addglobal float  NOISE_SYN_MEAN 0.                // synaptic noise mean
addglobal float  NOISE_SYN_RANGE 0.               // synaptic noise standard deviation

// setting input stimuli factors
addglobal float MOV_CELL {{{MY_NY}/2}*{MY_NX}+{EXC_CONN_DEPTH}+1}  // initialize moving dot
position
addglobal float S_RATE  0.               // input spiketrain rate

addglobal int D_SIZE                     // dot size for varing input rate
addglobal int D_SIZE_INH                 // dot size for varing input rate (inh)
```

```
addglobal float V_RATE_MEAN              // mean firing rate for sinusoidally changing rate
addglobal float V_RATE_AMP_E             // firing rate amplitude for sinusoidally changing
rate for E cells
addglobal float V_RATE_AMP_I             // firing rate amplitude for sinusoidally changing
rate for I cells
addglobal float V_RATE_FREQUENCY         // frequency for sinusoidally changing rate

float PI = 3.14159

// channel equilibrium potentials mV
// nominal rest potential used as a
float EREST_ACT = -70
                                         // reference potential for H-H channels
// actual resting membrane potential
float EREST = -70

// +55 mV
float ENa = 125.0 + EREST_ACT
// -80 mV
float EK = -10.0 + EREST_ACT
// 0 mV
float EEx = 70.0 + EREST_ACT
// -80 mV
float EInh = -10.0 + EREST_ACT

//  leakage potential used in place of EREST in soma
// -70 mV
float Eleak = EREST_ACT

// -58.3 mV
// float Eleak = 11.7 + EREST_ACT

// peak channel conductance      mS/synapse

// Excitatory
float GEx = 10.000       // mS/cm^2
// Inhibitory
float GInh = 5.369       // mS/cm^2

// Kohm-cm^2
float rm = 20.0          // 20,000 ohm cm^2
// uF/cm^2
float cm = 1.0           // 10e-6 (F cm-2)
// Kohm-cm
float ra = 0.2           //  200 ohm cm

// compartment dimensions (cm.)
addglobal float SOMA_L 15e-4
addglobal float SOMA_D 15e-4
```
